# The timing of death in acute pulmonary embolism patients regarding the mortality risk stratification at admission to the hospital

**DOI:** 10.1016/j.heliyon.2023.e23536

**Published:** 2023-12-12

**Authors:** Slobodan Obradovic, Boris Dzudovic, Piotr Pruszczyk, Ivica Djuric, Bojana Subotic, Jovan Matijasevic, Marija Benic, Sonja Salinger, Ljiljana Kos, Tamara Kovacevic-Preradovic, Irena Mitevska, Srdjan Kafedzic, Aleksandar Neskovic, Bjanka Bozovic, Nebojsa Bulatovic, Vladimir Miloradovic

**Affiliations:** a Clinic of Cardiology, Military Medical Academy of Belgrade, Serbia; b School of Medicine, University of Defense, Belgrade, Serbia; c Clinic of Emergency Internal Medicine, Military Medical Academy, Belgrade, Serbia; d Department of Internal Medicine and Cardiology, Medical University of Warsaw, Poland; e Institute of Pulmonary Diseases Vojvodina, Novi Sad, Serbia; f School of Medicine, University of Novi Sad, Serbia; g Clinic of Cardiology, Clinical Center Nis, Serbia; h School of Medicine, University of Nis, Serbia; i Clinic of Cardiology, Clinical Center Banja Luka, Bosnia and Herzegovina; j School of Medicine, University of Banja Luka, Bosnia and Herzegovina; k University Cardiology Clinic, Intensive Care Unit, Skopje, Macedonia; l Clinic of Cardiology, University Clinical Center Zemun, Serbia; m School of Medicine, University of Belgrade, Serbia; n Clinic of Cardiology, Clinical Center Podgorica, Montenegro; o School of Medicine, University of Podgorica, Montenegro; p Clinic of Cardiology, Clinical Center Kragujevac, Serbia; q School of Medicine, University of Kragujevac, Serbia

## Abstract

**Background:**

The management of patients with acute pulmonary embolism (aPE) depends on the severity of aPE. The timing of death in various aPE risk subgroups is only partially known.

**Methods:**

1618 patients with an objectively established aPE diagnosis with computed tomography pulmonary angiography enrolled in the regional PE registry were included in the study. According to ESC criteria, patients were stratified at admission to the hospital in four risk strata. The timing of PE-related and non-PE-related deaths was analyzed regarding mortality risk.

**Results:**

PE-related, and non-PE-related hospital death rates were 1.1 % and 1.5 % in low, 1.1 % and 4.8 % in intermediate-low, 8.1 % and 5.9 % in intermediate-high, and 27.7 % and 6.9 % in high-risk groups, respectively. The median time of PE-related and non-PE-related death across the PE mortality risk were: 4 (1.7–7.5) and 7.0 (4–14.7) days in low, 1.5 (1.0–9.5) and 11.5 (2.0–21.0) days in intermediate-low, 4.0 (2.0–9.0) and 9.0 (5.7–18.2) days in intermediate-high, 2.0 (1.0–4.75) and 7.0 (3.0–21.2) days in high-risk subgroups. 48.2 % and 17.1 % of patients who died in the high and intermediate-high risks died during the first hospital day. After the 6th hospitalization day, PE-related deaths were recorded in 43.9 % of deaths from intermediate-high and 17.9 % from high-risk subgroups.

**Conclusion:**

PE-related mortality is prominent on the first hospitalization day in high and intermediate-high-risk PE. A substantial proportion of intermediate-high and high-risk patient's PE deaths occurred after the first 6 days of hospitalization.

## 1 Introduction

According to the current guidelines [1] the initial treatment of acute pulmonary embolism (PE) depends on the patient's risk stratification at admission to the hospital. Patients who are hemodynamically compromised need urgent reperfusion therapy (high-risk PE), normotensive patients with right ventricle dysfunction and elevated cardiac troponin (cTn) and/or brain natriuretic peptide (BNP) blood levels represent an intermediate-high-risk stratum, and need monitoring in intensive care units, and others (intermediate-low and low-risk patients) may be considered for early discharge depending mostly on the management of comorbidities. Patients in the first category are at particularly high risk for early death, and prompt reperfusion therapy is considered to be lifesaving [1]. However, we do not know the exact proportion of high-risk patients who will actually succumb on the first hospital day. In patients with intermediate-high-risk PE, guidelines recommend close hemodynamic monitoring and reperfusion therapy in those who become hemodynamically unstable [1,2]. Some patients in this group will improve very soon after the introduction of supportive and anticoagulant therapy but a significant proportion of patients need more time and some of them even worsen in the first days from admission [[3], [4], [5]]. Generally, we do not know how much time we have to closely monitor intermediate-high-risk PE patients when is the most appropriate time to intervene and escalate therapy to prevent hemodynamic collapse, and when therapy will be much less efficient and with more adverse events.

The management of acute PE patients depends on the risk stratification of dying from PE recommended by the ESC guidelines from 2019. However, at least one-fourth of patients die from other causes, and acute PE and its treatment could contribute to premature death in various conditions. Hence, the timing of death in acute PE patients depends not only on the PE severity, but also on the complications related to anticoagulant treatment and hospitalization. Investigation of the timing to death in acute PE in respect of PE severity and the presence of comorbidities could be helpful to determine more precisely how long we need to monitor patients with various PE risk in intensive care units, and what kind of complications may arise, and when to expect these complications. Therefore, our study investigated the timing and proportion of patients with acute PE who died from pulmonary embolism and all-cause hospital death in relation to the European Society of Cardiology mortality risk stratification at admission.

## 2 Methodology

Data from this investigation was obtained from the Regional Pulmonary Embolism Registry (REPER) founded in 2015. Currently 5 university hospitals (Military Medical Academy Belgrade, Institute of Pulmonary Diseases Vojvodina, Clinical Center Nis, Clinical Center Kragujevac, Clinical Center Zemun) and one general hospital (GH Pancevo) from Serbia, and university hospitals - clinical centers from Banja Luka (Bosnia and Herzegovina), Podgorica (Montenegro) and Skopje (North Republic of Macedonia) recruited consecutively hospitalized PE patients for the registry. The registry started as the single center registry of hospitalized PE patients in 2012, and after 2015–2019 other institutions joined the Regional PE Registry.

### 2.1 Ethics statement

The study was approved, including the publication of the data derived from the registry, by the Ethic Committee of the Military Medical Academy, Belgrade, Republic of Serbia (code number of approval: 20/2022, 3/2021, 160/2019). The local ethical committees or other professional boards in charge allowed the participation of each center in the registry without public use of personal patient data. Patients gave their permission to participate in the registry.

All patients had confirmed diagnosis of PE by computed tomography pulmonary angiography (CTPA), or with visualized thrombus in the right heart on admission echocardiography. Symptoms characteristic of PE patients or worsening chronic symptoms which can be explained by acute PE were presented for no more than 14 days before the admission.

According to European Society of Cardiology PE guidelines from 2019, patients were stratified into the 4 risk strata upon admission [1,6]. Patients who were resuscitated, or were in shock (persistent systolic arterial blood pressure less than 90 mmHg) because of massive PE were considered as high-risk PE; normotensive patients with signs of RV dysfunction on echocardiography or CTPA with elevated blood levels of cardiac troponin were classified as intermediate-high risk; normotensive patients with RV dysfunction, but without elevated blood levels of cardiac troponin represented intermediate-low risk PE, and those who had no hypotension and RV dysfunction were low-risk patients.

Simplified pulmonary embolism severity index (sPESI) was also calculated at admission for all patients according to the proposed criteria [7].

The timing of death and the cause of death were recorded in the REPER database. Two variables represented the cause of death. The first was a variable where doctors classified the death as PE-related or non-PE-related death and the second-string variable was where they can freely record the diagnosis which was adduced as the main cause of death in the medical history.

### 2.2 Endpoints

The primary endpoint of this study is timing in days from the hospital admission to hospital PE-related death, or non-PE-related death [1,8]. Patients, whose death was PE-related, had either sudden cardiac arrest without any other reasons except PE and/or signs of obstructive shock. Shock due to PE was defined if the patient had severe systolic arterial blood hypotension (<90 mmHg) persisted even under vasopressors, or patients had significant blood pressure fall from the admission values (>40 mmHg) without any other possible explanation. Other possible causes of shock such as hemorrhagic or septic shock, hypovolemia, and low cardiac output because of heart diseases were excluded during the diagnostic work-up.

Non-PE related death were deaths where there was established other, very likely dominant cause of death. Attending physicians who take care of patients recorded the first diagnosis in medical history which is the most probably main cause of death, and if it was not PE, we considered this cause as non-PE related death. The list of non-PE-related death diagnoses in respect of PE severity was presented in Table 2. The hospital day of death is also defined and recorded in the database.

**Table 1:** Baseline characteristics of patients in the REPER registry regarding the intra-hospital survival status and the main cause of death.

	Survivals N = 1441	PE-related death N = 107	Non-PE related death N = 70	p
Age – y, SD	63, 16	69, 15	71, 12	<0.001
Females – n, %	765, 53.1	59, 55.1	40, 57.1	0.748
Spontaneous PE – n, %	770, 53.5	40, 43.9	22, 31.4	<0.001
PE after surgery – n, %	220, 15.3	14, 13.1	12, 17.1	0.748
Malignancy – n, %	181, 12.6	18, 16.8	18, 25.7	0.004
COPD – n, %	142, 9.9	13, 12.1	16, 22.9	0.002
CHF – n, %	192, 13.3	23, 21.5	23, 32.9	<0.001
Coronary disease – n, %	152, 10.7	20, 19.4	11, 16.2	0.013
Stroke – n, %	89, 6.2	17, 15.9	9, 13.0	<0.001
Diabetes type 2 – n, %	270, 18.7	32, 29.9	20, 29.0	0.003
Obesity – n, %	256, 21.3	18, 20.7	10, 16.9	0.721
GFR^1^ – n, %	454, 31.7	74, 69.2	40, 58.0	<0.001
GFR^2^ – n, %	93, 6.5	27, 25.5	18, 26.1	<0.001
PE severity – n, %				
sPESI> 0	952, 66.1	98, 91.6	64, 91.4	<0.001
Low-risk	516, 35.8	6, 5.6	8, 11.4	<0.001
Intermediate-low risk	356, 24.7	4, 3.7	18, 25.7	
Intermediate-high risk	437, 30.3	41, 38.3	30, 42.9	
High risk	132, 9.2	56, 52.3	14, 20.0	
Thrombolytic therapy	353, 24.5	47, 43.9	15, 21.4	<0.001
Initial anticoagulant therapy				
UFH (bolus + infusion)	500, 84.7	61, 10.3	29, 4.9	<0.001^3^
LMWH	889, 91.4	46, 4.7	38, 3.9	
Fondaparonux	21, 95.5	0, 0.0	1, 4.5	
Rivaroxaban	17, 100.0	0, 0.0	0, 0.0	

Y – years, SD – standard deviation, COPD – chronic obstructive pulmonary disease, CHF – chronic heart failure, GFR^1^ –glomerular filtration rate <60 ml/min, GFR^2^ –glomerular filtration rate <30 ml/min, sPESI – simplified pulmonary embolism score index, UFH – unfractionated heparin, LMWH – low molecular weight heparins. ^3^P value is calculated with Hi square test only for UFH and LMWH.

**Table 2:** PE-related and non-PE-related hospital mortality across the 4 stratums of the mortality risk model with a median number of days from the hospital admission to death and the interquartile range.

Mortality risk	PE related death	Non PE related death
Low risk (N = 530)		
Mortality	6 (1.1 %)	8 (1.5 %)
Time of death in days – median (25th-75th)	4.0 (1.75–7.5)	7 (4.0–14.75)
Intermediate-low risk (N = 378)		
Mortality	4 (1.1 %)	18 (4.8 %)
Time of death in days – median (25th-75th)	1.5 (1.0–9.5)	11.5 (2.0–21.0)
Intermediate-high risk (N = 508)		
Mortality	41 (8.1 %)	30 (5.9 %)
Time of death in days – median (25th-75th)	4.0 (2.0–9.0)	9.0 (5.75–18.25)
High-risk (N = 202)		
Mortality	56 (27.7 %)	14 (6.9 %)
Time of death in days – median (25th-75th)	2.0 (1.0–4.75)	7.0 (3.0–21.25)

### 2.3 Statistics

In this study, patients were divided into three subgroups, those who survived, the patients who died very probably due to acute PE, and those who died predominantly from other causes, so-called non-PE related death. The differences between patients' characteristics across three subgroups of patients are tested by the student's T-test and Chi-square test. The frequencies of hospital PE-related death and other causes-related death from each day from day 1–5, and cumulative from the 6–30 days are presented as numbers of patients and percentages across the mortality risk stratums and presented as figures. The median time of PE-related death and other causes of death with interquartile ranges are also presented. The differences between the median times from death in respect of mortality risk were calculated with the Mann-Whitney *U* test and presented as p values in the text. For the significance of difference, we considered p value less than 0.05.

## 3 Results

The basic characteristics of patients regarding survival status and the main cause of death are presented in Table 1. There were several important differences between PE patients who survived and those who died of PE-related causes, or of other causes. Survivors were much younger than the deceased, both in the group with PE-related death and in the patients who died for other causes. A history of chronic heart failure, chronic obstructive pulmonary disease, and malignancy in the last 6 months was more prevalent in patients who died from other causes than in patients who survived or died from PE. The history of coronary heart disease, stroke, and diabetes was slightly more often presented in patients with PE-related death than in patients who died from other causes, but both of them were more often present compared to the survivor's group. Glomerular filtration rate of less than 60 ml/min was more frequent in patients who died from PE, slightly lower in patients who died from other causes, and much lower in survivors. As it is expected the PE-related mortality rate was higher in patients who were treated with thrombolytic therapy and unfractionated heparin.

The simplified PESI score was higher than 0 in more than 90 % of patients who died from PE and in almost the same percentage in patients who died from other reasons.

The main causes of non-PE-related death with respect to ESC mortality risk stratification are presented in Table 1 in the supplemental material. The most common diagnosis as the first cause of death was sepsis (17.1 %), pneumonia (17.1 %), acute renal failure without hemodynamic compromise (11.4 %), progression of malignant disease (10.0 %), and acute heart failure (10.0 %). Some of these diagnoses could represent the differential diagnosis with acute PE, and probably acute PE contributes to death at least in intermediate-high and high-risk PE patients. However, on the other side, those patients who were classified as non-PE related death had at least very serious comorbidities which are for sure important factors for death outcomes.

**Fig. 1:**
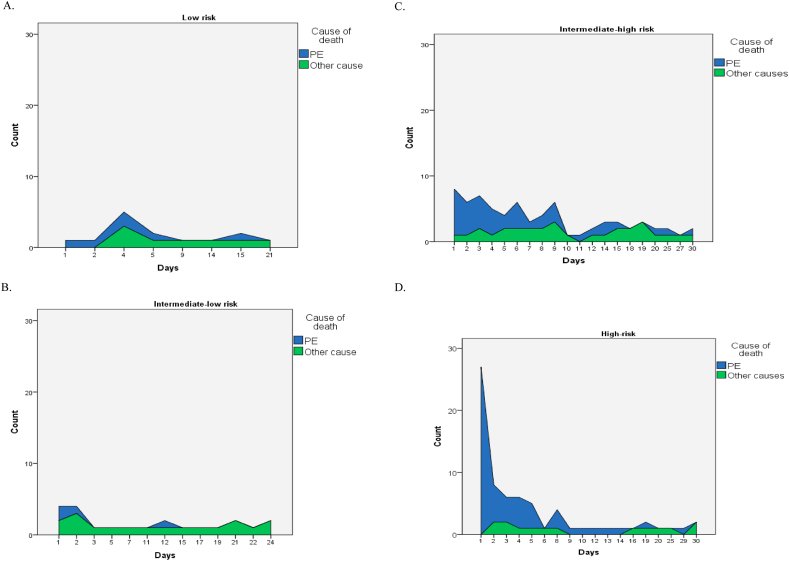
A–D. PE-related and non-PE-related hospital death in the low (A), intermediate-low (B), intermediated-high (C), and high-risk PE patients. A.

PE-related and other cause-related hospital mortality and the median time in days from hospital admission to death are presented in Table 2 and Fig. 1 A-D. Hospital mortality from PE was low in low-risk and intermediate-low-risk groups (1.1 % for both) and the median time from hospitalization to death was 4.0 (1.75–7.5) and 1.5 (1.0–9.0) days, respectively. In the intermediate-high- and high-risk PE, mortality rates were 8.1 % and 27.7 %, respectively, with significantly different timing of death between these groups, 4.0 (2.0–9.0) days vs 2.0 (1.0–4.75) days, respectively, p = 0.002.

Non-PE-related death in hospitals is higher compared to PE-related death in the low and intermediate-low subgroup of patients with a much longer median time from hospital admission to death outcome. In the intermediate-high subgroup of patients, non-PE-related death was presented in 5.9 % of patients. In this subgroup of patients, non-PE-related death occurred significantly later in the course of hospitalization than PE-related death (median time in days was 9.0 for non-PE-related death vs 4.0 days for PE-related death, p = 0.001). In the high-risk group, the difference between PE-related death and non-PE-related death was very pronounced 27.7 % vs 6.9 %, with significantly shorter hospital time for PE-related death patients (median time in days is 2.0 vs 7.0 days, p < 0.001) (Table 2). The distribution of all-cause death and PE-related death across the ESC risk groups is depicted in Supplementary Fig. 1 (panels A and B).

Among the whole group of patients who died, on days 2, 3, and 4 of hospitalization, PE-related death was 10.7 %, 7.1 %, and 8.9 % in the high-risk PE, and 12.2 %, 12.2 %, and 9.8 % in the intermediate-high risk patients. Additionally, in the cohort of PE-related death patients, 17.9 % of high-risk, and 43.9 % of intermediate-high-risk patients died in hospital from PE after the 6th day of hospitalization (Table 3).

**Table 3:** Daily PE-related and non-PE-related hospital death in the first 5 days from the hospital admission and after the 6th day across the mortality risk subgroups.

Mortality risk	N (% of patients who died)					
	1st day	2nd day	3rd day	4th day	5th day	6th-30th day
Low risk (N = 530)						
Non-PE-related death	0	0	0	3 (37.5)	1 (12.5)	4 (50.0)
PE related death	1 (16.7)	1 (16.7)	0	2 (33.4)	1 (16.7)	1 (17.7)
Intermediate-low (N = 378)						
Non-PE-related death	2 (11.1)	3 (16.7)	1 (5.6)	0	1 (5.6)	11 (61.1)
PE-related death	2 (50.0)	1 (25.0)	0	0	0	1 (25.0)
Intermediate-high (N = 508)						
Non-PE-related death	1 (3.3)	1 (3.3)	2 (6.7)	1 (3.3)	2 (6.7)	23 (76.7)
PE-related death	7 (17.1)	5 (12.2)	5 (12.2)	4 (9.8)	2 (4.9)	18 (43.9)
High (N = 202)						
Non-PE-related death	0	2 (14.3)	2 (14.3)	1 (7.1)	1 (7.1)	8 (57.1)
PE related death	27 (48.2 %)	6 (10.7)	4 (7.1)	5 (8.9)	4 (7.1)	10 (17.9)

Nineteen out of 71 (26.8 %) patients who died in the intermediate-high group and 37 out of 70 (52.8 %) patients who died in the high-risk group were treated with thrombolytic therapy. The use of thrombolysis did not appear to be associated with the timing of death in either intermediate-high (median time to death 6.0 [22.0–9.0] with thrombolysis vs 6.5 [3.0–14.75] days without thrombolysis; p = 0.345), or in high-risk PE (median time 2.0 [1.0–7.0] vs 3.0 [1.0–6.75] days, respectively; p = 0.716).

## 4 Discussion

The management of acute PE depends on the PE severity which can be easily assessed by the ESC model of PE-related mortality risk using simple clinical and echocardiography parameters together with cardiac troponin measurement at the admission to hospital [1]. Therefore, patients are classified into 4 risk categories, where the high-risk patients are those with severe arterial hypotension who have a hospital mortality rate of more than 20 %, intermediate-high-risk patients are normotensive and have right ventricle dysfunction with elevated blood levels of cardiac troponins, and mortality rate between 5 and 10 %, intermediate-low risk patients who have RV dysfunction without blood cardiac troponin elevation and low-risk patients who have neither of this represents the risk categories where acute PE is rarely a cause of hospital death. Patients with high-risk acute PE are at high risk of early death, during the first hours from the symptom onset, and immediate reperfusion therapy is recommended for this subgroup of patients. In patients classified into the intermediate-high risk PE parenteral anticoagulation with close hemodynamic monitoring is recommended and they should receive thrombolysis if they deteriorate [[1], [2], [3], [4], [5]]. However, the proportion of patients who died early in these two most severe PE subgroups and the more precise timing of death are not well investigated. There are some important results from this investigation that could be useful for the proper management of acute PE patients with a higher risk. Importantly, according to our “real world” data, almost 50 % of deaths in high-risk PE patients occurred in the first 24 h from hospital admission. Moreover, in the intermediate high-risk group, 17.7 % of deaths also occurred in the first 24 h, however, after that time there are about 10 % of deaths per day till day 5. These findings imply several important conclusions. High-risk patients need urgent pharmacological or mechanical reperfusion, and a considerable number of intermediate-high patients also need fast decisions for applying reperfusion. Interestingly, 43.9 % and 17.9 % of PE-related death happened after the 5th hospital day in intermediate-high- and high-risk subgroups which mean that these patients have a much longer risk for deterioration than recommended 48–72 h of monitoring in the intensive care facilities. It is obvious that more proper continuous risk assessment is needed, especially for the intermediate-high-risk subgroup.

The timing of death in aPE has not been thoroughly investigated. In the study of Heit et al., PE-related mortality on the first day of presentation was 23.5 %, without a precise relation between the hemodynamic state and death [9]. We did not find any data in the literature concerning the timing of death in intermediate-high-risk PE patients or patients who are normotensive at admission. The proper timing of reperfusion therapy may be crucial for the outcome of severe acute PE. Rawal et al. [10] have shown that patients undergoing early (in the first 48 h from admission) catheter-directed therapy with ultrasound-facilitated thrombolysis had greater benefit than later (after the 48 h) application of this therapy. In concordance with this, Beydilli et all [11], although in the small cohort of 49 high-risk PE patients, also suggested that early treatment with systemic thrombolysis is important, and even a small delay of 5 min, may represent the increased risk for PE related death. For high-risk patients, it is obviously very important to treat the patients with reperfusion therapy as early as possible since our results also confirmed that the first-day PE-related mortality of 48.2 % in this subgroup was very high during this short period.

It is worth underlining that intermediate high-risk PE patients who deteriorated despite initial anticoagulation had almost 50 % PE-related mortality even when rescue thrombolysis was applied [12], this indicates that treatment should be escalated in some of these patients before hemodynamic instability develops and the “wait and watch strategy” could be fatal if we wait for the hemodynamic collapse for the reperfusion therapy. Instead of this, we need more sophisticated risk estimation for the prediction of deterioration in intermediate high-risk PE patients.

Death rate regarding the ESC mortality risk assessment model was similar in our study to the studies of Beccatini et al. and Cugno et al. where hospital mortality was 22 % and 40 % in high-risk, 7.7 % and 14.8 % in the intermediate-high-risk, 6.0 % and 9.4 % in the intermediate-low risk and 0.5 % and 2.5 % in the low-risk group, respectively [6,13]. However, in our study, we separated PE-related death from non-PE-related causes of death, but cumulative death rates are similar to those two studies. The treatment strategy for acute PE must be oriented toward PE-related death risk factors. However, in some circumstances, it is very difficult to determine the main cause of death even with an autopsy. Concomitant PE at least contributes to all other causes of death in many cases.

The data about the timing for treatment failure for either patient treated with thrombolytic therapy or anticoagulant therapy are scarce. In the cohort of 488 patients [5] who underwent thrombolysis 8.2 % did not improve 36 h after thrombolysis and needed rescue reperfusion. In the PEITHO randomized trial, 5 % of patients who were on anticoagulant therapy deteriorated and have to proceed to reperfusion, mostly in the first 72 h of hospital admission [4]. The pulmonary embolism study group showed that the calculation of the PESI_48_ (sPESI score after 48 h) is an accurate method for assessing early treatment response in intermediate high-risk patients. The score identifies 8 % of patients with acute PE with very low death risk during the first month after discharge. However, the mortality of patients who were reclassified as higher risk 48 h after the diagnosis of PE (classes IV and V) was as high as 50 % of patients. These patients should be closely observed and monitored in ICU units during that period since this group of patients might have an additional benefit from thrombolysis [14].

We previously presented that resolution of S waves in aVL and DI ECG leads in high-risk and intermediate-high-risk patients during the first five days (but it can be used much earlier at the time of resolution happened) of treatment is a simple method to define patients with low risk for PE related mortality [15].

Our data are concordant with these observations that the increased risk for deterioration may persist in a certain number of high and intermediate-high-risk patients, much longer than 48–72 h, which is the recommended time for close monitoring of these patients by the guidelines, and the significant number of them still not improve enough to be released from the intensive care units in that period of time. In our study, almost 43.9 % of PE-related deaths happened after the sixth hospitalization day. Therefore, it is necessary to carefully estimate the risk from an individual patient and when is it appropriate time to transfer them from intensive care to the ward, where there is no such level of medical monitoring.

## 5 Limitations

There are several significant limitations of this study. Since this is a registry, the patient's cause of death was not centrally adjudicated, and because of the complexity of patients, it is doubtful if patients died from PE, or if PE significantly contributed to the death or not. However, our sub-investigators classified the death as PE-related in the case of sudden death, and death after some period of shock state with or without inotropic stimulation, but without other possible reasons for that, like the ESC guidelines defined high-risk PE. For patients who were admitted with the features of high-risk PE, it is better to have time determined in hours or even minutes, than days, since approximately half of them have died during the first hospitalization day. The timing of thrombolytic therapy did not record in this study, but it will be very useful to have these data, especially in the correlation of various clinical, biomarkers, and echocardiography parameters to the outcome of intermediate-high risk patients who deteriorate.

## 6 Conclusion

PE-related mortality is prominent in the first hospitalization day in both high and intermediate high-risk PE patients, and it remains high during the next four days. A substantial proportion of intermediate-high and high-risk patient deaths from PE occur after the first 6 days of hospitalization. The repeated determination of the National Early Warning Score [16], serial measurements of biomarkers, ECG, and echocardiography parameters could be used for the selection of patients with high-risk and intermediate-high-risk PE who can be transferred from the intensive care unit to a semi-intensive or ordinary ward.

## Data availability statement

Data associated with this study has not been deposited into a publicly available repository. However, data will be available on request.

## CRediT authorship contribution statement

**Slobodan Obradovic:** Writing – review & editing, Writing – original draft, Supervision, Methodology, Data curation, Conceptualization. **Boris Dzudovic:** Writing – review & editing, Investigation, Formal analysis, Data curation. **Piotr Pruszczyk:** Writing – review & editing, Investigation, Formal analysis, Data curation. **Ivica Djuric:** Investigation, Formal analysis, Data curation. **Bojana Subotic:** Investigation, Formal analysis, Data curation. **Jovan Matijasevic:** Investigation, Formal analysis, Data curation. **Marija Benic:** Investigation, Formal analysis, Data curation. **Sonja Salinger:** Investigation, Formal analysis, Data curation. **Ljiljana Kos:** Investigation, Formal analysis, Data curation. **Tamara Kovacevic-Preradovic:** Investigation, Formal analysis, Data curation. **Irena Mitevska:** Investigation, Formal analysis, Data curation. **Srdjan Kafedzic:** Investigation, Formal analysis, Data curation. **Aleksandar Neskovic:** Writing – review & editing, Visualization, Supervision, Investigation, Formal analysis, Data curation. **Bjanka Bozovic:** Investigation, Formal analysis, Data curation. **Nebojsa Bulatovic:** Investigation, Formal analysis, Data curation. **Vladimir Miloradovic:** Investigation, Formal analysis, Data curation.

## Declaration of competing interest

The authors declare that they have no known competing financial interests or personal relationships that could have appeared to influence the work reported in this paper.
