# Supplementary material for: The timing of death in acute pulmonary embolism patients regarding the mortality risk stratification at admission to the hospital
Source: Heliyon. 2023 Dec 12;10(1):e23536. doi: 10.1016/j.heliyon.2023.e23536 (PMC10767379; doi:10.1016/j.heliyon.2023.e23536)

Supplementary figure 1. Kaplan-Meier curves regarding ESC model of mortality risk for all-cause death (Panel A) and PE-related hospital death (Panel B). Log rank test for both comparisons is p<0.001.

A


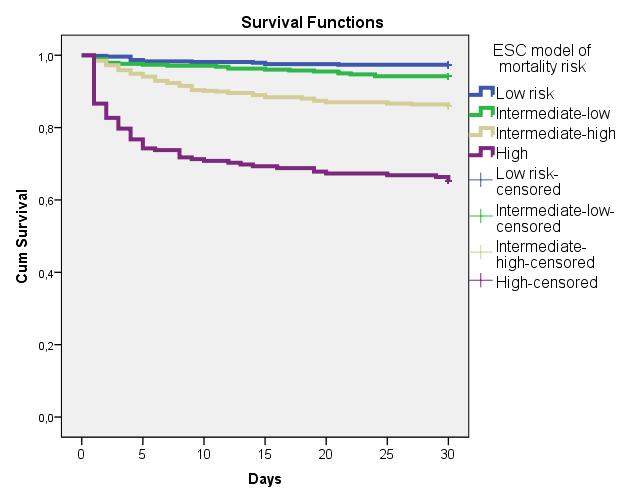


B


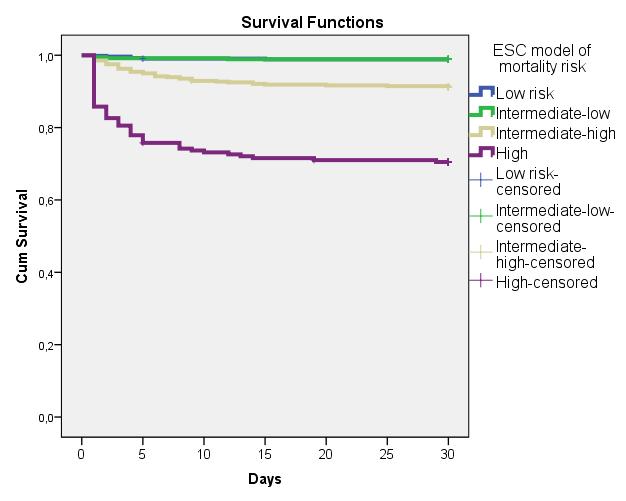

Supplement: Multimedia component 1 [file mmc1.docx]
