# Supplementary material for: The timing of death in acute pulmonary embolism patients regarding the mortality risk stratification at admission to the hospital
Source: Heliyon. 2023 Dec 12;10(1):e23536. doi: 10.1016/j.heliyon.2023.e23536 (PMC10767379; doi:10.1016/j.heliyon.2023.e23536)
Supplement: Multimedia component 2 [file mmc2.docx]

Supplementary material:

Table S1. The causes of death in the subgroup non-PE related death.

| Cause of death | Low risk | Intermediate-low risk | Intermediate-high risk | High risk | Total |
| --- | --- | --- | --- | --- | --- |
| Malignant disease  Intracranial hemorrhage  Gastrointestinal bleeding  West-Nile meningitis  Acute heart failure  Sepsis  Pneumonia  Worsening COPD or asthma  Acute renal failure with MOF  Acute respiratory distress  Cerebral infarction  Clostridium colitis  COVID-19 pneumonia  Neuroleptic syndrome  Hemolytic uremic syndrome  Acute myocardial infarction  DIC  Total | 1  1  0  1  2  0  0  0  1  0  0  1  1  0  0  0  0  8, 11.4% | 3  1  1  0  2  0  4  1  3  0  1  0  0  0  0  1  1  18, 25.7% | 3  0  0  1  2  8  7  1  4  1  1  0  0  1  1  0  0  30, 42.9% | 0  1  1  0  1  4  1  0  0  1  3  0  1  0  1  0  0  14, 20.0% | 7, 10.0%  3, 4.3%  2, 2.9%  2, 2.9%  7, 10.0%  12, 17.1%  12, 17.1%  2, 2.9%  8, 11.4%  2, 2.9%  5, 7.1%  1, 1.4%  2, 2.9%  1, 1.4%  2, 2.9%  1, 1.4%  1, 1.4%  70, 100.0% |

^1^Five out of 7 patients have had a history of heart failure with reduce or mildly reduce ejection fraction. ^2^COPD – chronic obstructive pulmonary disease, ^3^All patients with acute renal failure did not have hypotension and shock, MOF – multi-organ dysfunction, ^6^DIC - Disseminated intravascular coagulation.
